# Mitochondria and Viral Infection: Advances and Emerging Battlefronts

**DOI:** 10.1128/mbio.02096-21

**Published:** 2022-01-25

**Authors:** Mahsa Sorouri, Tyron Chang, Dustin C. Hancks

**Affiliations:** a Department of Immunology, University of Texas Southwestern Medical Center, Dallas, Texas, USA; b Genetics, Disease, and Development Graduate Program, University of Texas Southwestern Medical Center, Dallas, Texas, USA; Ohio State University; Ohio State University

**Keywords:** mitochondria, virus, mimics, virologs, apoptosis, pyroptosis, mitochondrial dynamics, OXPHOS, TCA cycle, MAVS, DAMP, mtDNA, mtdsRNA, mtROS, MISTR, interferon, supercomplexes, NDUFA4, C15orf48, micropeptides

## Abstract

Mitochondria are dynamic organelles vital for energy production with now appreciated roles in immune defense. During microbial infection, mitochondria serve as signaling hubs to induce immune responses to counteract invading pathogens like viruses. Mitochondrial functions are central to a variety of antiviral responses including apoptosis and type I interferon signaling (IFN-I). While apoptosis and IFN-I mediated by mitochondrial antiviral signaling (MAVS) are well-established defenses, new dimensions of mitochondrial biology are emerging as battlefronts during viral infection. Increasingly, it has become apparent that mitochondria serve as reservoirs for distinct cues that trigger immune responses and that alterations in mitochondrial morphology may also tip infection outcomes. Furthermore, new data are foreshadowing pivotal roles for classic, homeostatic facets of this organelle as host-virus interfaces, namely, the tricarboxylic acid (TCA) cycle and electron transport chain (ETC) complexes like respiratory supercomplexes. Underscoring the importance of “housekeeping” mitochondrial activities in viral infection is the growing list of viral-encoded inhibitors including mimics derived from cellular genes that antagonize these functions. For example, virologs for ETC factors and several enzymes from the TCA cycle have been recently identified in DNA virus genomes and serve to pinpoint new vulnerabilities during infection. Here, we highlight recent advances for known antiviral functions associated with mitochondria as well as where the next battlegrounds may be based on viral effectors. Collectively, new methodology and mechanistic insights over the coming years will strengthen our understanding of how an ancient molecular truce continues to defend cells against viruses.

## INTRODUCTION

Mitochondria are double membrane-bound organelles that are involved in several facets of cell biology essential for viability. Uniquely, this organelle is suspected to have originated more than two billion years ago when an alphaproteobacterium, an aerobic prokaryote, was engulfed by an archaeon cell. Contemporary mitochondria are well characterized as cellular hubs for critical metabolic processes and reactions including fatty acid (FA) oxidation, the tricarboxylic acid (TCA) cycle, and oxidative phosphorylation (OXPHOS) (1, 2). Mitochondrial metabolic programs are generally thought to maintain tissue homeostasis across cell types and organisms. Predictably, alterations in mitochondrial metabolic programs often occur in response to stress, such as changes in nutrient availability. Along with metabolism, mitochondria are synonymous with the execution of cell death as a means to control infections and increasingly appreciated as signaling platforms for immunity (3).

Over recent years, numerous reports have highlighted the essential roles for mitochondria in host defense during viral infections. A now classic example, which has been intensely studied, involves the mitochondrial antiviral-signaling (MAVS) protein (4–7). MAVS functions as a key adaptor molecule to transduce signals from upstream pattern-recognition receptors (PRRs), like retinoic acid-inducible gene I (RIG-I) (8), that detect RNAs from viruses to trigger type I interferon signaling (IFN-I). However, whether other dimensions of mitochondrial biology are linked to antiviral responses is less resolved. To shed light on emerging interfaces, we review new aspects further implicating the mitochondrial regulation of cell death, signaling, metabolic reprogramming, and dynamics as tipping points during viral infection. Our discussion is presented in the context of established battlegrounds and antagonism of the mitochondrial arsenal by classic and newly identified viral factors.

## ACTIVATION OF MITOCHONDRIAL CELL DEATH PATHWAYS DURING VIRAL INFECTIONS

**FIG 1:**
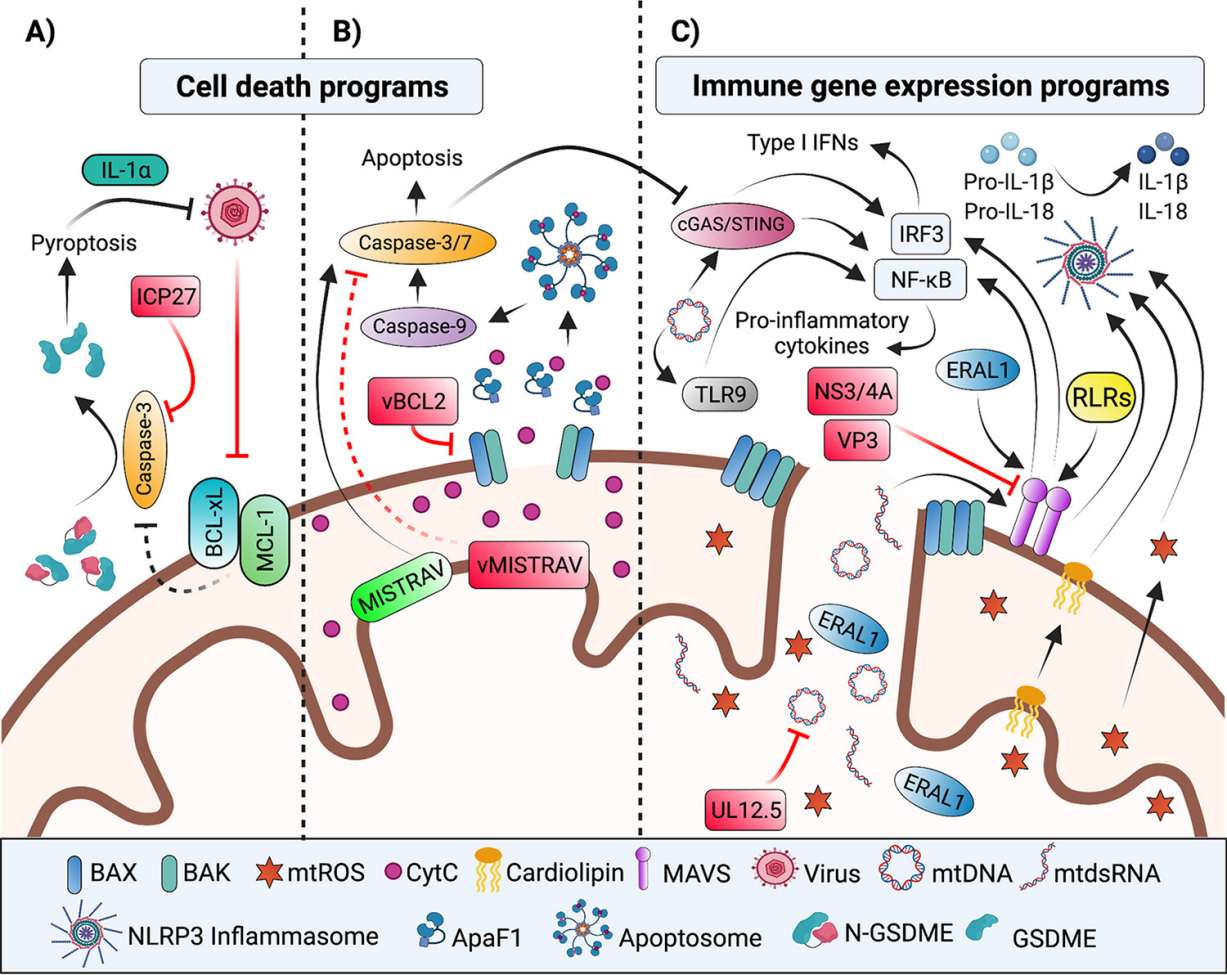
Host–virus interfaces shaping mitochondrial battlegrounds of cell death and immune signaling. Mitochondrial activities linked to the regulation of antiviral defenses and/or antagonized by viral factors (red) are shown. (A) Virus-induced inhibition of protein synthesis leads to downregulation and inactivation of anti-apoptotic BCL-2 family members MCL-1 and BCL-xL, respectively. The ensuing mitochondrial damage culminates in caspase-3 cleavage of GSDME and pyroptosis (40). The HSV-1 ICP27 protein prevents GSDME-mediated pyroptosis by inhibiting caspase-3 through an undefined mechanism (40). (B) The mitochondrial pathway of apoptosis is initiated by internal stress cues like viral infection. These stress cues stimulate proapoptotic BCL2 proteins in a series of events that ultimately lead to MOMP. MOMP allows for cytoplasmic release of several mitochondrial proteins including CytC. Interaction of CytC with Apaf-1 results in apoptosome assembly and activation of the caspase cascade. vBCL2 virologs counteract apoptosis by inhibiting host proapoptotic BCL2 proteins (24). MISTRAV, an interferon inducible ETC accessory factor, regulates the apoptotic response, while its virolog squirrelpox-encoded vMISTRAV is antiapoptotic (52). (C) Mitochondria activate antiviral nuclear gene expression programs via either MAVS signaling or release of resident mitochondrial DAMPs. PAMP or DAMP recognition by RLRs leads to activation of MAVS, which ultimately induces the activation and nuclear translocation of transcription factors NF-κB and IRF3 and 7 (not shown) to upregulate the expression of proinflammatory cytokines as well as type I or III interferons (not shown), respectively. Viral-encoded proteins antagonize MAVS function. For example, the HCV NS3/4A protein blocks IFN induction by cleaving MAVS (63). In addition, the rotavirus VP3 induces phosphorylation and thereby proteasomal degradation of MAVS (65). Protrusion of the IMM through OMM pores generated during MOMP allows for release of immunogenic mitochondrial matrix components, such as mtDNA, mtdsRNA, and ERAL1. While mtdsRNA (106) and ERAL1 (107) induce MAVS signaling, mtDNA release activates the cGAS/STING pathway (74, 75), the NLRP3 (93–96) and AIM2 (not shown) (75, 97) inflammasome pathways, and TLR9 (91) signaling. Importantly, caspase-3 cleavage of cGAS and IRF3 regulates whether MOMP leads to inflammatory signaling or apoptosis (81–83). Viral-encoded factors antagonize the mtDNA response. For example, the HSV-1 UL12.5 protein promotes mtDNA depletion (76, 103). In addition to mtDNA, other components of damaged mitochondria, such as cardiolipin (99), or mitochondrial metabolic by-products, such as mtROS (177), induce NLRP3 inflammasome activation and subsequent production of inflammatory cytokines, IL-1β and IL-18. mtDNA induction of the TLR9 pathway activates NF-κB (91), while activation of the cGAS/STING pathway results in nuclear translocation of NF-κB and IRF3 (74, 75), to regulate inflammatory gene expression. BCL-2, B-cell lymphoma-2; MCL-1, myeloid cell leukemia-1; BCL-xL, BCL extra-large; GSDME, gasdermin E; MOMP, mitochondrial outer membrane permeabilization; CytC, cytochrome c; Apaf-1, apoptotic protease activating factor-1; MISTR, MItochondrial STress Response; MISTRAV, MISTR antiviral; ETC, electron transport chain; MAVS, mitochondrial antiviral signaling; PAMP, pathogen associated molecular pattern; DAMP, damage associated molecular pattern; IMM, inner mitochondrial membrane; OMM, outer mitochondrial membrane; ERAL1, E. coli Ras-like 1; NLRP3, NOD-, LRR, and pyrin domain-containing 3; AIM2, absent in melanoma 2; TLR9, toll-like receptor 9. Figure not drawn to scale. Figure created with BioRender.com.

Cell death is an evolutionarily conserved means to restrict viral replication and protect the host organism (9–13). Death of an infected cell disrupts the viral replication cycle while also serving as a strategy to alert various cells (e.g., immune cells) of the infection (14–17). Diverse programmed cell death pathways (Fig. 1A and B) (18) have been characterized; with apoptosis, necroptosis, and pyroptosis all having documented roles in managing viral infections (19). Of established cell death pathways, apoptosis displays the most known overlap with mitochondria.

Apoptosis is carried out by caspases, which are cysteine-aspartic proteases initially synthesized as inactive procaspases (20). Activation of the extrinsic or intrinsic apoptotic pathway leads to caspase activation, which is achieved by self-cleavage or cleavage by other caspases at aspartic acid residues. The extrinsic pathway is initiated by external stress signals (e.g., TNF-α, FASL) that are sensed by death receptors on the plasma membrane (e.g., TNF-α receptor, Fas) (18, 21). In contrast, the intrinsic route—also referred to as the mitochondrial pathway—of apoptosis is initiated by internal stressors like viral infection.

Activation of intrinsic apoptosis is regulated by a balance between the pro- and antiapoptotic members of the B-cell lymphoma-2 (BCL-2) family of proteins. Internal stress cues can stimulate pro-apoptotic BCL-2 proteins, such as BCL-2-interacting mediator of cell death (BIM) (14, 16). Anti-apoptotic BCL-2 proteins like BCL-2, BCL extra-large (BCL-xL) and myeloid cell leukemia-1 (MCL-1) can inhibit the progression of apoptosis by counteracting the activated proapoptotic proteins (14). Apoptosis ensues when antiapoptotic proteins are saturated or absent. In such instances, the activated proapoptotic proteins bind to two other proapoptotic proteins: BCL-2-associated X protein (BAX) and BCL-2 antagonist/killer (BAK). This leads to oligomerization of BAK and BAX on the outer mitochondrial membrane (OMM), which drives the formation of macropores in a process known as mitochondrial outer membrane permeabilization (MOMP) (Fig. 1B) (14, 15). MOMP facilitates the cytoplasmic release of several mitochondrial proteins, including cytochrome c (CytC). Binding of CytC to the scaffold protein apoptotic protease activating factor-1 (Apaf-1) in the cytoplasm promotes the formation of a supramolecular complex known as the apoptosome (Fig. 1B) (14, 18). The apoptosome activates the initiator caspase-9. Caspase-9 subsequently cleaves the executioner procaspases (caspases 3, 6, and 7) to unleash their activity and a cascade of destruction: cleavage of the cytoskeleton and the ensuing membrane blebbing, nuclear and DNA fragmentation, as well as demolition of other organelles including the Golgi apparatus, the endoplasmic reticulum (ER), and the mitochondrial network in preparation for phagocytosis (Fig. 1B) (14, 22).

A valuable signature illuminating essential host activities in immune defense, like apoptosis, is the identification of viral-encoded antagonists of specific cellular functions. Chiefly, the premise is based on the limited coding capacity of viruses. Namely, viruses are restricted to only encoding and maintaining what may be deemed required. Indeed, many viruses encode factors that counteract apoptosis through diverse strategies, one of which is mimicry (23). Viral mimicry is frequently achieved via the repurposing of cellular genes acquired by horizontal gene transfer during infection, factors referred to as virologs.

Viral mimics of BCL2 proteins appear to have evolved independently in several double-stranded DNA virus lineages. For instance, viral BCL2 homologs (vBCL2) are encoded in the genomes of poxviruses, herpesviruses, iridoviruses, and asfarviruses (23, 24). vBCL2 proteins typically resemble antiapoptotic BCL2 factors and inhibit proapoptotic BCL2 proteins (Fig. 1B). These factors have been intensely studied to understand the regulation of apoptosis in response to infection. Virologs also serve as valuable tools to illuminate mechanisms of their cellular counterparts. A well-studied example is vaccinia virus (VACV) F1L, a BCL2 homolog, which interacts with BAK to inhibit BAK/BAX activation (25).

While BCL2-mediated release of mitochondrial effectors is largely associated with apoptosis, new data suggest this protein family may also be linked to pyroptotic cell death. Pyroptosis is a type of lytic cell death (26, 27), which is a defined response to viral (28, 29) and bacterial infection (30), executed by the gasdermin (GSDM) family proteins (31, 32). This pathway is initiated by PRRs like NOD-like receptors (NLRs) (28, 33) and absent in melanoma 2 (AIM2) (34) that detect various pathogen associated molecular patterns (PAMPs) (e.g., flagellin, dsDNA) and danger-associated molecular patterns (DAMPs). This sensing results in the formation of a large, multi-protein complex termed the inflammasome. The inflammasome consists of the PRR, an adaptor protein named apoptosis-associated speck-like protein containing a caspase recruitment domain (ASC), and (pro)caspase-1 (35). Here, caspase-1 becomes active by cleaving itself then targeting key inflammatory cytokines like interleukin-1 beta (IL-1β) and interleukin-18 (IL-18) as well as gasdermin D (GSDMD). Proteolytic processing of GSDMD licenses cell lysis mediated by this factor (32).

Underscoring the importance of pyroptosis in host defense, viruses encode modulators of this cell death program. For example, the 3C protease of enterovirus 71 (EV71) degrades GSDMD during infection in 293T cells (36). On the other hand, encephalomyocarditis virus (EMCV) activates the NOD-, LRP, and pyrin domain-containing 3 (NLRP3) inflammasome (37) by stimulating Ca^2+^ influx via its viroporin 2B protein (38). Relatedly, it has been shown that translation inhibition, a common response to viral infection (39), in keratinocytes triggers a signaling cascade that results in GSDME-mediated pyroptosis (Fig. 1A) (40). Specifically, virus-mediated translational inhibition results in a decrease of MCL1, an anti-apoptotic BCL2 factor, and inactivation of BCL-xL. As a result, caspase-3 is activated, which processes GSMDE to its active form. Importantly, the same study demonstrated that herpes simplex virus (HSV-1) ICP27 counteracts this pathway (Fig. 1A). Caspase-3 activation is presumed to be induced via the canonical pathway involving mitochondria; however, future studies will likely clarify this as well as any notable differences. An additional link between mitochondrial functions and pyroptosis is the modulation of GSDMD-mediated pore formation by mitochondrial reactive oxygen species (mtROS) (41). In contrast, data are lacking for major cross talk between mitochondria and necroptosis (42) notwithstanding several reports of viral-encoded antagonists (43–46). Nevertheless, mitochondrial activities provoked by invading viruses overlap with other regulated cell death pathways, and the critical nature of these interactions are accentuated by viral-encoded antagonists.

While viral and cellular mechanisms regulating BCL2 factors and steps downstream of MOMP are documented in detail, whether viruses directly antagonize mitochondrial functions inside the organelle during cell death is less clear. The number of viral proteins that enter mitochondria are few, but some have been identified. A well-defined example is influenza A PB1-F2 which localizes to inner mitochondrial membrane (IMM). PB1-F2 induces apoptosis (47, 48) and antagonizes other mitochondrial functions like MAVS signaling (49). Data suggest that PB1-F2 induces apoptosis by impacting the mitochondrial permeability transition pore (mPTP) complex through interactions with voltage dependent anion channel (VDAC) and adenine nucleotide translocator 3 (ANT3). This pro-apoptotic function is hypothesized to be proviral by killing off immune cells (50). Interestingly, the host can use NLRX1, a member of the NOD-like receptor family, to counteract PB1-F2 in macrophages to control influenza A virus infection (51).

A newer battleground involves virologs of related, nuclear-encoded electron transport chain (ETC) accessory factors, which are micropeptides (<100 amino acids), encoded by divergent viruses (52). The related host factors are ultraconserved and largely uncharacterized, and comprise the MItochondrial STress Response (MISTR) circuit. The host MISTR factors are differentially regulated by stress signals including cytokines and hypoxia (52). Interestingly, MISTR micropeptides are the first ETC complex factors reported to be stolen by viruses. However, components linked to the TCA cycle have been identified recently in viral genomes and discussed further in the next section (53, 54).

MISTR factors reside in the IMM and peripherally associate with ETC complexes (55–59). Deletion of human MISTR AntiViral (MISTRAV, previously C15orf48), which is induced by immune signals, attenuates chemical- and virus-induced apoptosis (Fig. 1B). In contrast, loss of the paralog that is downregulated by stress (MISTR1 also known as NDUFA4) increases apoptotic responses by the same triggers. MISTRAV has a virolog encoded by squirrelpox virus (vMISTRAV), and MISTR1 has a virolog encoded by a DNA virus that infects sturgeon (vMISTR1) (52). Furthermore, a MISTR virolog is encoded by a giant virus that infects algae (vMISTRA). Key support for MISTR factors playing essential but undefined functions in cell death stem, in part, by data showing that vMISTRAV also attenuates virus-induced apoptosis (Fig. 1B). Complementing these findings, a subsequent study showed that cellular MISTRAV/C15orf48 and MISTR1/NDUFA4 regulate Complex IV activity in IL-1β -treated cells (59), activities that are implicated in control of the inflammatory response. The mechanism for how MISTR factors regulate ETC complexes as well as other function(s) is currently unclear. We hypothesize that MISTR factors are foreshadowing key roles for ETC composition in shaping host defenses. Of particular interest are the formation and regulation of higher-order stoichiometric arrangements of the ETC termed supercomplexes (SC) (60). One possibility is that the MISTR factors may alter ETC complexes to remodel cristae (61), a process important for the release of CytC in apoptosis (62).

## MITOCHONDRIAL REGULATION OF IMMUNE GENE EXPRESSION PROGRAMS DURING VIRAL INFECTION

Mitochondrial activities induce nuclear gene expression programs important for antiviral defense. Specifically, signaling can be triggered by interactions occurring on the mitochondrial surface and the release of DAMPs (e.g., mtDNA) due to volatile alterations of mitochondrial morphology. MAVS is the prototype for antiviral signaling at the mitochondrial surface. Given MAVS is instrumental for IFN-I signaling during RNA virus infection, several virus-encoded proteins that directly antagonize MAVS functions have been characterized. For example, hepatitis C virus (HCV) NS3/4A, a serine protease, localizes to mitochondria, where it cleaves MAVS and thus blocks IFN-I induction (Fig. 1C) (63). Consistent with being a determinant of infection outcomes, MAVS displays strong signatures of rapid evolution in primate genomes, which includes positively selected sites proximal to the NS3/4A protease target site shown to impact cleavage (64).

In addition to HCV, other viruses encode mitochondrial proteins that can degrade MAVS. For instance, the rotavirus RNA capping enzyme, VP3, localizes to mitochondria, where it induces phosphorylation and subsequent proteasomal degradation of MAVS (Fig. 1C) (65). Whether other cellular factors transduce signals to alter immune gene expression, either by shuttling to mitochondria during infection or as permanent residents at the organelle like MAVS anchored to the OMM, is an area of interest. Potential candidates may be host factors that are both regulated by IFN-I and have evidence for mitochondrial localization (e.g., predicted mitochondrial localization signal sequence or biochemical support from a resource such as MitoCarta (66, 67), a compendium of mitochondrial proteins).

Along with signal transduction on the surface, release of molecules from within mitochondria results in gene expression changes. Prominent, emerging agonists are mtDNA and mtdsRNA. When these nucleic acids are leaked from mitochondria, they are sensed as DAMPs by proteins that stimulate antiviral gene expression programs. Documented means for the release of mtDNA involve the mPTP (68, 69), VDAC pores (70), and BAX/BAK-mediated pores. The latter represents the most well-studied avenue. Expansion of BAX/BAK mediated pores that drive MOMP during apoptosis permits extrusion of the IMM through the OMM. The subsequent rupture of the IMM allows matrix components including mtDNA and mtdsRNA to escape from mitochondria (71–73). Extra-mitochondrial mtDNA activates several DNA-sensing antiviral pathways like cGAS/STING signaling to upregulate IFN-I (Fig. 1C) (74–76). Indeed, infection with several different viruses has been reported to induce mtDNA release. Notably, the explanation for the counterintuitive observation that RNA viruses stimulate DNA sensors cGAS/STING is, in part, through the release of mtDNA, which in turn can control the infection (77–80).

Two outcomes of MOMP during infection, apoptosis versus mtDNA induction of IFN-I, are regulated by caspase-3 cleavage of cGAS and IRF3 (Fig. 1B and C) (81–83). Consistently, minority MOMP or “sub-lethal” apoptosis (84–86) induced by viruses, intracellular bacteria, and a protozoan parasite triggers cytokine secretion and inflammation in the absence of a cell death phenotype (87, 88). These immune responses are linked to sensing of cytostolic mtDNA by cGAS/STING. mtDNA release is also controlled by resident mitochondrial factors. Tfam is the multifunctional, mitochondrial transcription factor (89). Haploinsufficiency of *Tfam* (+/-) in MEFs results in IFN-I induction that is dependent on cGAS/STING (76). Notably, the mtDNA/cGAS signaling in the TFAM mutant can be attenuated using dideoxycytidine (ddC), a nucleoside analog and an antiviral drug that reduces mtDNA copy number and nucleoid size. The aforementioned defines packaging of mtDNA as a regulator of its release (76). Interestingly, viral factors also have been shown to drive mtDNA release. Specifically, influenza virus M2 and EMCV 2B protein both exhibit viroporin activity that promotes mtDNA release in infected cells (90). mtDNA is also detected by PRRs that induce inflammatory signaling. Examples include cytoplasmic PRRs, such as NLRs and absent in melanoma-like receptors (ALRs), as well as membrane-bound PRRs such as toll-like receptors (TLRs) (91, 92). Here, cytoplasmic mtDNA induces the activation and assembly of the NLRP3 (93–96) and AIM2 (75, 97) inflammasomes. Consequently, caspase-1 is activated, which in turn processes pro-IL-1β and pro-IL-18 to facilitate their secretion. Additional cross talk in mitochondrial immune signaling is demonstrated by a role for MAVS in NLRP3 inflammasome-mediated production of IL-1β (98) via recruitment of this complex to mitochondria. Likewise, translocation of cardiolipin from the IMM to the OMM is essential for NLRP3 inflammasome recruitment and activation (Fig. 1C) (99).

TLR9 is another PRR that senses extra-mitochondrial mtDNA to initiate inflammatory signaling (91). Following detection of PAMPs and DAMPs, TLR signaling through the p38 and mitogen activated protein kinase (MAPK) pathways induces ΝF-κΒ signaling and ΝF-κΒ target genes (Fig. 1C) (92, 100). The physiological relevance of this signaling axis is highlighted by activation of TLR9 signaling by mtDNA in the bloodstream in severe trauma patients (92). In this case, mtDNA released due to injury activates p38 MAPK enzyme via TLR9 in human neutrophils. The canonical substrate of TLR9 is unmethylated cytosine-phosphate-guanine (CpG) dinucleotides, which is common to bacteria and DNA viruses (101). Distinctly, dengue virus (DENV), which has an RNA genome, also activates TLR9 signaling in human dendritic cells (DCs) due to mtDNA release during the infection (102). Here, the release of mtDNA is linked to reduced binding of TFAM to mtDNA because of increased PKA-mediated phosphorylation of TFAM (102).

Underscoring mtDNA as a battlefront in infection are emerging instances of viral factors that antagonize this genome. A unique example is the amino-terminal truncated isoform of the HSV-1 UL12 protein, known as UL12.5. UL12.5 localizes to mitochondria and promotes mtDNA depletion (Fig. 1C). The mechanism appears independent of UL12.5 nuclease activity (76, 103), but associated with an ability to modulate mitochondrial nucleases endonuclease G (ENDOG) and exo/endonuclease G (EXOG) (104). Another antagonist again stems from a herpesvirus. In this case, the Epstein-Barr virus (EBV) DNA-binding protein, Zta, co-opts the mitochondrial single-stranded binding (mtSSB) protein, through direct binding, and relocalizes it to the nucleus, where the genome of this virus resides (105). This interaction is important for EBV lytic replication as well as reducing mtDNA genome replication. While this list is currently quite short, this field is relatively young but moving very quickly in terms of cytostolic mtDNA-evoked immune responses. Presumably, due to the diversity of viruses that cause mtDNA release and the extent of downstream PRRs that recognize it, several new antagonists will soon be uncovered.

Along with mtDNA, mtdsRNA released into the cytosol is sensed by PRRs. Indeed, melanoma differentiation-associated protein 5 (MDA5) detection of cytostolic mtdsRNA, which accumulates during MOMP in a BAX/BAK-dependent manner, leads to IFN-I expression through MAVS signaling (Fig. 1C) (106). Notably, while cytostolic mtdsRNA is present in cell lines and in patients, its abundance is determined by a sensitized background where polynucleotide phosphorylase (PNPase) is dysfunctional. Forthcoming studies will surely shed light on the role and regulation of this DAMP in host defense.

The release of proteins from within mitochondria can also augment immune signaling. Namely, release of the mitochondrial matrix protein E. coli Ras-like 1 (ERAL1) during RNA virus infection by BAX/BAK enhances MAVS signaling *in vitro* and *in vivo* during RNA virus infection (Fig. 1C) (107). This ERAL1 activity is RIG-I dependent. ERAL1 is a GTPase in the mitochondrial matrix, which is required for the proper assembly of the 28S small mitochondrial ribosomal subunit, involved in cell viability (108). ERAL1 potentiates MAVS/IFN-I induction by at least two mechanisms. First, ERAL1 promotes TRIM25-mediated K63 ubiquitination of RIG-I and MDA-5. Second, ERAL1 directly interacts with MAVS, which promotes MAVS oligomerization and seeds the formation of MAVS prion-like structures important for antiviral signaling (109, 110). Consistently, ERAL1 knockdown in THP-1, HT-29, HEK293T, and HeLa cells attenuates IFNβ production during RNA virus infection. A physiological role for ERAL1 is apparent as *Eral^+/-^* mice are more susceptible to RNA virus infection-associated lethality than the control *Eral^+/+^* mice (107).

## ALTERATIONS TO MITOCHONDRIAL MORPHOLOGY DURING VIRAL INFECTION

Mitochondria are dynamic organelles subject to frequent cycles of fusion, defined as joining of mitochondria, and fission, defined as fragmentation of mitochondria (111, 112). Fusion and fission are important for mitochondrial associated functions and homeostasis (112). Mitochondrial fusion is regulated by mitofusin1 (MFN1), MFN2, and optic atrophy 1 (OPA1) (113). In contrast, mitochondrial fission is regulated by dynamin related protein 1 (DRP1) (114). Fission is connected to mitochondrial turnover, which is regulated by a process termed mitophagy (115). Mitochondrial fusion, fission, and turnover largely comprise mitochondrial dynamics.

Mitophagy is a selective form of autophagy, a catabolic process mediated by lysosomal degradation and recycling of intracellular macromolecules (115). Defective mitophagy is linked to neurodegenerative diseases (116–120) including Parkinson’s disease (PD) (121–123). In the absence of mitophagy, the persistence of damaged mitochondria leads to activation of the cGAS/STING pathway and an inflammatory phenotype (123). These findings suggest an anti-inflammatory role for mitophagy.

**FIG 2:**
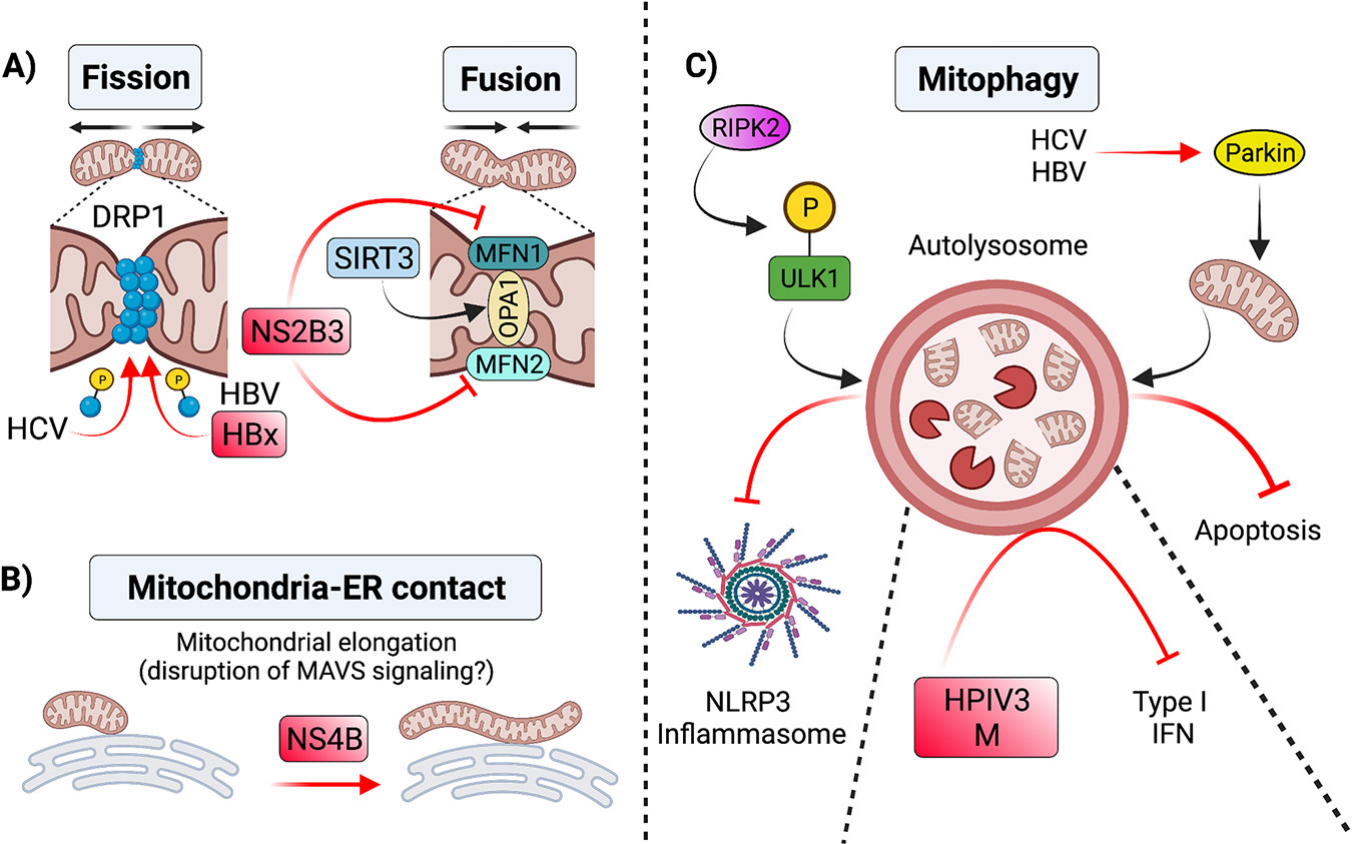
Alterations to mitochondrial morphology during viral infection. (A) Changes in mitochondrial dynamics during viral infection have antiviral effects. For instance, SIRT3-mediated activation of OPA1 during HCMV infection promotes mitochondrial fusion, which interferes with viral replication (124). In contrast, viral infections can shift mitochondrial dynamics to favor replication and suppress innate defenses. For example, HCV (128) and HBV (129) induce phosphorylation and recruitment of DRP1 to mitochondria to enhance mitochondrial fission and subsequent mitophagy. Increased mitophagy driven by both viruses suppresses apoptosis and promotes viral persistence (128, 129). Dengue virus NS2B3 protein cleavage of MFN1/2 impairs mitochondrial fusion and inhibits type I IFN signaling (130). (B) Viruses induce changes in the interactions of mitochondria with other organelles that impact mitochondrial dynamics. For instance, dengue virus NS4B induces mitochondrial elongation, which alters MAM structure and dampens the immune response (134). (C) Mitophagy in antiviral responses. During influenza A infection in mice, RIPK2 mediated phosphorylation of ULK1 induces Parkin-independent mitophagy, which negatively regulates NLRP3 inflammasome activation. HCV and HBV also promote mitophagy by inducing translocation of Parkin to mitochondria (128, 129), which favors viral persistence by mitigating apoptosis. The M protein of the HPIV3 is also involved in mitophagy induction of infected cells, which results in inhibition of IFN-I response (136). SIRT3, sirtuin 3; OPA1, optic atrophy A1; HCV, hepatitis C virus; HBV, hepatitis B virus; DRP1, dynamin related protein 1; MAM, mitochondria-associated membrane; ULK1, Unc-51 like autophagy activating kinase 1; MFN1, mitofusin 1; MFN2, mitofusin 2; RIPK2, receptor interacting protein kinase 2; HPIV3, human parainfluenza virus. Figure not drawn to scale. Figure created with BioRender.com.

Several effectors of mitochondrial dynamics shape infection outcomes through direct protein–protein interactions. For instance, OPA1 interaction with mitochondrial deacetylase sirtuin 3 (SIRT3) promotes mitochondrial fusion, which suppresses human cytomegalovirus (HCMV) viral production (Fig. 2A) (124). Deletion of the mitochondrial fusion regulators MFN1 and MFN2 abrogates the MAVS-IFN-I signaling pathway during viral infection (125). Interaction between MFN1 and MAVS was reported using coimmunoprecipitation (126). A subsequent study demonstrated that this interaction promotes redistribution of MAVS to speckle-like patterns on mitochondria and impacts MAVS signaling in response to viral infection and transfected 5’ppp-RNA (127).

The characterization of viral factors antagonizing mitochondrial dynamics and host effectors of these processes reinforces mitochondrial morphology as an emerging battleground. For instance, HCV promotes fission in Huh7 cells by inducing phosphorylation of DRP1 at S616, a key posttranslational modification important for DRP1 recruitment to mitochondria (128). HCV-induced fission is followed by mitophagy that attenuates apoptosis (Fig. 2A) (128). These alterations are associated with enhanced viral secretion and suppressed IFN synthesis. Similarly, transfection of Huh7 cells with either the full hepatitis B virus (HBV) genome or the HBV master regulator, HBx, also triggers fission by inducing S616 phosphorylation of DRP1 and thus mitochondrial translocation (Fig. 2A) (129). Markedly, mitochondrial fusion is antagonized by cleavage of MFN1 and MFN2 by the DENV NS2B3 protease (Fig. 2A). This cleavage impairs IFN-I RLR signaling (130).

Mitochondria do not function in isolation, and interactions with other organelles such as the ER have reported roles during infection. Stable contacts between mitochondria and the ER are critical for many cellular processes, including lipid synthesis, Ca^2+^ signaling, mitochondrial division, and metabolism (131). Specific subdomains of mitochondrial-ER junctions, termed mitochondria-associated membrane (MAM), are critical in induction of MAVS signaling (110). MAMs have established roles in mitochondrial dynamics (132, 133). Evidence comes from DENV NS4B, which induces mitochondrial elongation via inactivation of DRP1 (Fig. 2B). The resulting disruption in MAMs dampens the antiviral immune response (134). Promoting mitochondrial elongation may be a common strategy shared among flaviviruses as Zika virus displays a similar activity.

Clearance of mitochondria by mitophagy is another emerging battlefront. During mitophagy, mitochondria that are damaged beyond repair via fission and fusion are recycled. Specifically, damaged mitochondria are “tagged” with autophagosomal markers to form autophagosomes, which fuse with a lysosome to destroy cargo. Influenza infection in mice is controlled via negative regulation of NLRP3 inflammasome activation by parkin-independent mitophagy. The effector of parkin-independent mitophagy, receptor interacting protein kinase 2 (RIPK2), triggers mitophagy by phosphorylation of Unc-51 Like Autophagy Activating Kinase 1 (ULK1) (Fig. 2C). Consistently, *Ulk1*^−/−^ mice infected with influenza virus display exacerbated immunopathology as well as increased IL-18 production and caspase 1 activation (135).

Viral induction of mitophagy is common in infected cells. Viruses such as HCV and HBV trigger mitophagy by inducing translocation of Parkin to mitochondria. Mitophagy in these infected cells attenuates apoptosis and thus allows for persistent viral infection (Fig. 2C) (128, 129). Direct binding of host mitophagy effectors by viral proteins to counteract antiviral responses has been described. The matrix protein (M) of human parainfluenza virus type 3 (HPIV3) binds a receptor for mitophagy initiation, Tu translation elongation factor mitochondrial (TUFM), to induce Parkin-independent mitophagy in HeLa cells. By an unclear mechanism, M-induced mitophagy leads to inhibition of IFN-I response (Fig. 2C) (136). As battlegrounds emerge that mold mitochondrial morphology, it is tempting to speculate that future studies may reveal themes related to “altered self,” which serve to indirectly sense pathogen infection at other organelles (137) and function as defense mechanisms predicted by the guard hypothesis (40, 138, 139).

## CHANGES IN MITOCHONDRIAL METABOLISM DURING VIRAL INFECTIONS

Metabolism has exploded as an important regulator of immunity, especially functions characteristic of immune cells discussed below, an area termed immunometabolism (140). Cells leverage a variety of metabolic programs to survive including glycolysis, fatty acid oxidation, the TCA cycle, and OXPHOS. Some immune cells are stable in their metabolic activity. Immune cells like IL-4-stimulated macrophages (141) and induced regulatory T cells (Tregs) (142, 143) rely on OXPHOS for energy production to support their anti-inflammatory and immunosuppressive functions. In contrast, during interferon-γ-mediated polarization, M1 macrophages shift from OXPHOS to glycolysis (144), whereas inhibition of glycolysis in macrophages favors a shift toward a M2-like state (145). Antigen-activated T helper cells also undergo a similar shift to launch rapid effector T-cell responses (146).

**FIG 3:**
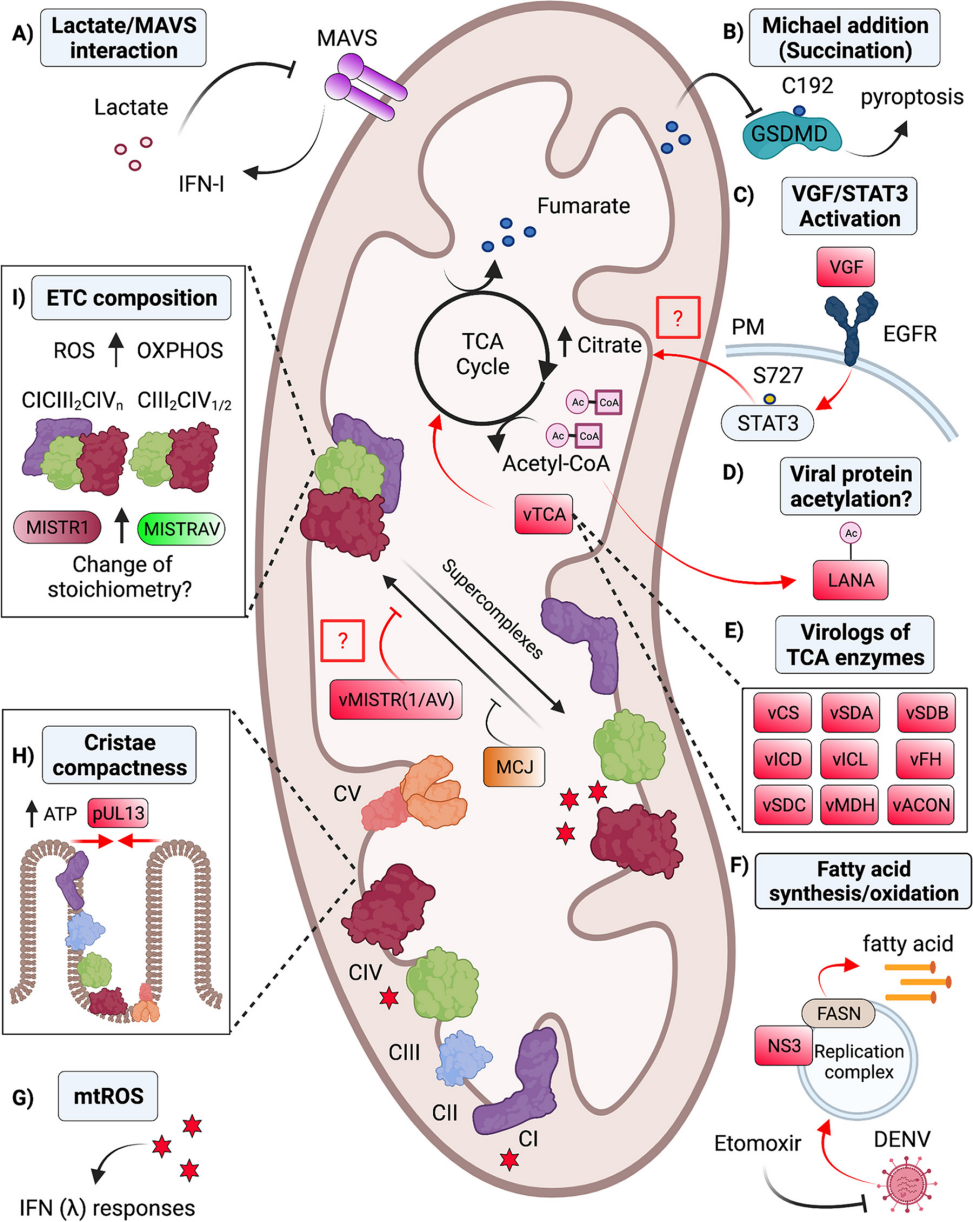
Mitochondrial metabolic pathways are textbook functions which impact the outcomes of viral infection. Host–virus interfaces overlapping with mitochondrial metabolism. Viral-encoded antagonists and viral mediated activities shown in red. (A) Glycolytic metabolites can negatively regulate antiviral signaling. Lactate suppresses IFN-I signaling through a direct interaction with MAVS (147). (B) TCA intermediates promote host defense. Fumarate represses pyroptosis via a nonenzymatic chemical modification (Michael addition) to GSDMD in mouse macrophages (154). (C) VACV rewires TCA metabolism. VGF derived from VACV binds to EGFR to induce STAT3 activation via phosphorylation at serine 727. Through an unknown mechanism, activation of STAT3 increases citrate levels (159). (D) TCA intermediates serve as regulators of viral effectors. Acetyl-CoA is important for the acetylation of KSHV LANA proteins (161). (E) Viruses (e.g., NCLDV) also encode homologs of enzymes from the TCA cycle (vTCA), which likely manipulate the abundance of TCA intermediates (53). (F) DENV antagonizes fatty acid synthesis. DENV NS3 protein recruits FASN to sites of viral replication to increase fatty acid synthesis (163). In agreement, chemical inhibition of fatty acid oxidation using etomoxir inhibits dengue virus replication (165). (G) OXPHOS by-products fine-tune antiviral signaling. mtROS regulates the IFN-λ response through an unclear mechanism (179). (H) HCMV pUL13 modulates OXPHOS to increase ATP production and the compactness of cristae (182). (I) Virologs for MISTR micropeptides indicate ETC composition represents an emerging battlefront during infection. Host MISTRAV and MISTR1 functions may modulate ETC complexes and supercomplexes to shape host defenses. vMISTRAV and vMISTR1 likely counteract these responses (52). Furthermore, host proteins such as MCJ can inhibit the formation of SCs and repress antiviral defense (189). TCA, tricarboxylic acid; OXPHOS, oxidative phosphorylation; MAVS, mitochondrial antiviral-signaling protein; GSDMD, gasdermin D; VGF, viral growth factor; VACV, vaccinia virus; EGFR, epidermal growth factor receptor; STAT3, signal transducer and activator of transcription 3; FASN, fatty acid synthase; KSHV, Kaposi's sarcoma-associated herpesvirus; MCJ, methylation-controlled J protein; MISTR, MItochondrial STress Response; mtROS, mitochondrial reactive oxygen species; NCLDV, nucleocytoplasmic large DNA viruses; SCs, supercomplexes; PM, plasma membrane; vTCA, viral homologs of TCA enzymes. Figure not drawn to scale. Figure created with BioRender.com.

Rapid metabolic adaptions that modulate early antiviral defenses are less understood. Recently, lactate has been shown to directly bind MAVS to suppress IFN-I production (Fig. 3A) (147). Lactate is a glycolytic by-product produced by lactate dehydrogenase A (LDHA). Consistently, chemical inhibition and genetic deletion of LDHA enhance restriction of multiple RNA viruses both *in vitro* and *in vivo* (147). These data suggest that metabolites may be important regulators of the early antiviral response.

More defined are major changes in metabolism occurring during viral infection (148). Upregulation of different aspects of glycolysis, including glycolytic rates, can favor viral replication. A shift to glycolysis is likely beneficial to the virus as it overlaps with the pentose phosphate pathway, which facilitates viral nucleotide synthesis (149). Additional evidence stems from studies inhibiting glycolysis during infection. Use of the nonhydrolysable glucose analog 2-deoxyglucose, which inhibits phosphoglucoisomerase, significantly reduces rhinovirus and norovirus replication (150). However, detailed mechanisms of how viruses establish preferred metabolic environments during infection is largely unknown. Still, insights may come from human adenovirus E4ORF1. E4ORF1 binds MYC to induce transcription of glycolysis-associated genes in epithelial cells. Consistently, a mutant E4ORF1 is unable to enhance MYC-transcriptional activation of glycolytic genes (151).

While much attention has been given to glycolysis in immune defense, areas of mitochondrial metabolism like the TCA cycle are ripe for discovery (152). The TCA cycle, which operates in the mitochondrial matrix, is driven by acetyl-CoA produced from pyruvate generated by glycolysis. TCA cycle metabolites may serve as substrates to bolster defenses, as demonstrated by the following: (i) acetyl-CoA is a histone acetylase co-factor essential for effector T-cell IFN-γ expression (153), (ii) fumarate inhibits pyroptosis in macrophages by reacting with GSDMD via Michael addition reaction (Fig. 3B) (154), and (iii) immune-responsive gene 1 (IRG1) conversion of cis-aconitate to itaconate induces expression of anti-inflammatory genes by nuclear factor erythroid 2-related factor 2 (Nrf2) to counter pro-inflammatory responses (155). Both fumarate and itaconate can potently restrict viral replication by unknown mechanisms. Addition of fumarate restricts Severe Acute Respiratory Syndrome Coronavirus 2 (SARS-CoV-2) in Vero cells (156), whereas itaconate restricts a broad spectrum of viruses, including VACV and HSV-1, in HaCaT cells (157).

Underscoring the TCA cycle as a key interface during viral infection, proviral effects of modulating TCA cycle homeostasis have been characterized. HCMV elevates TCA intermediates in fibroblast and epithelial cells as evidenced by changes detected using metabolomic analysis combined with isotope-labeled carbon atoms from ^13^C-glucose labeling (158). Distinctly, intracellular citrate levels increase when human foreskin fibroblasts (HFFs) are infected with VACV (159). The increases in citrate require VACV viral growth factor (VGF). During VACV infection, VGF induces epidermal growth factor receptor (EGFR) signaling to activate the noncanonical signal transducer and activator of transcription 3 (STAT3) pathway (Fig. 3C). Consistently, infection with VACVΔVGF fails to result in active STAT3 (phospho-S727). In agreement, the increases in citrate are blocked by STAT3 and EGFR inhibitors. These findings show that the VGF/EGFR/STAT3 pathway regulates citrate levels by an unclear mechanism during VACV infection. Furthermore, viruses can co-opt TCA cycle metabolites to serve as donors for posttranslational modification of viral proteins. For instance, acetyl-CoA can serve as the substrate for acetylation of latency-associated nuclear antigen (LANA) encoded by Kaposi's sarcoma-associated herpesvirus (KSHV) (Fig. 3D). Lysine acetylation of LANA is needed to maintain the viral latency-lytic cycle (160, 161).

Strikingly, diverse nucleo-cytoplasmic large DNA viruses (NCLDV), which include giant viruses, have independently acquired not only specific TCA enzymes but also different combinations of TCA enzymes (Fig. 3E) (53). Notably, some mimiviruses encode almost all the TCA enzymes. Virologs for the following TCA enzymes have been reported in a recent metagenome analysis focused on NCLDVs: citrate synthase (CS), aconitate (ACON), isocitrate dehydrogenase (ICD), isocitrate lyase (ICL), succinate dehydrogenase subunits A, B, and C (SDA, B, C), fumarate hydratase (FH), and malate dehydrogenase (MDH). Phycodnaviridae encodes SDB and SDC. Iridoviridae only encodes ICL. These data indicate extensive, yet unexplored biology for the TCA cycle during viral infection.

Yet why do viruses target the TCA cycle? Citrate, which is increased by VACV, is also involved in FA synthesis. FA synthesis produces fatty acids derived from acetyl-CoA as well as other TCA intermediates. Reasonably, viruses may rewire the TCA cycle to increase FA synthesis to generate lipids like very long chain fatty acids required to produce membranes for virions (162). For example, DENV NS3 recruits fatty acid synthase (FASN) from the cytosol to sites of viral replication (Fig. 3F). This relocalization seems important to increase production of lipid molecules essential for formation of the replication complex for assembly of virions (163).

Viruses can also perturb fatty acid oxidation, which occurs in the mitochondrial matrix. Fatty acid oxidation, also known as β-oxidation, is a catabolic process where fatty acids are broken down to generate energy. One study shows that fatty acid oxidation is critical to support the growth of measle virus (MV) (164). Specifically, siRNA knockdown of enoyl-CoA hydratase—an enzyme in the β-oxidation pathway—suppresses MV replication via an unknown mechanism (164). Another investigation found that infection with DENV induces degradation of lipids and enhances β-oxidation using radiolabeled hydrogen atoms from ^3^H-palmic acid (165). Furthermore, treatment with the β-oxidation inhibitor etomoxir decreases DENV replication (Fig. 3F) (165). In summary, viruses hijack fatty acid oxidation to facilitate viral growth, but the mechanisms by which viruses alter fatty acid oxidation to subvert host defenses are not fully defined.

Perhaps more infamous than the TCA cycle and fatty acid oxidation in mitochondrial metabolism is the electron transport chain. The ETC mediates OXPHOS to produce ATP. Given that viruses target fundamental cellular circuitry, it may not be surprising that the ETC is in the crosshairs during infection (166). Residing in the IMM, the OXPHOS system consists of five multisubunit ETC complexes (CI-V) and two small electron carriers (CytC and ubiquinone) (167, 168) that transfer electrons from NADH and FADH_2_ to reduce O_2_ to water. As electrons are transferred between the ETC complexes, CI, III, and IV pump protons from the mitochondrial matrix into the intermembrane space (IMS). This process generates an electrochemical gradient that fuels the catalysis of ATP from ADP and phosphate by ATP synthase (Complex V) (169–171). The seminal finding linking the ETC to host defense was the identification of CytC as the factor released from mitochondria to trigger apoptosome formation (172, 173). As discussed above, viruses commonly suppress CytC release by antagonizing host proapoptotic BCL2 factors using mimicry. Additional alterations to the ETC, such as changes in activity, structure, and by-product levels in response to infections by pathogens have been reported and are discussed in detail below.

ROS are well-known ETC by-products (174) that can have detrimental effects like protein and nucleic acid damage. Increasingly, ROS is appreciated for its function in signaling (175). A relevant example is ROS regulation of immune defenses against viruses and other pathogens (176). The cellular sources of ROS include NADPH oxidase and mitochondrial respiration, with mtROS primarily produced by CI and CIII of the ETC (166). A defined contribution of mtROS to innate immunity is the activation of inflammasomes, such as NLRP3 (177). In addition to the role of mtROS in immune signaling, mtROS has been implicated in other defenses during infection through undefined mechanisms (175, 178). One study shows that suppression of mtROS with a superoxide scavenger, in the context of influenza virus infection in human nasal epithelium, represses type III IFN signaling and increases viral replication (Fig. 3G) (179). Further evidence for antiviral effects of mtROS and cross talk among immune responses stems from the mitophagy regulator Parkin. Parkin is a ubiquitin ligase that restricts mtROS production. Specifically, bone marrow-derived macrophages (BMDMs) from Parkin-deficient mice exhibit higher clearance of viral replication via the mtROS-NLRP3 axis (180).

Changes in ETC activity are thought to precede MOMP during viral infection (181). Whether relevant changes occur in ETC activity independent of virus-induced cell death or at earlier stages in infection is less well understood. At least one viral protein has been shown to modulate ETC activity. HCMV pUL13 localizes to mitochondria and alters ETC activity and mitochondrial architecture (Fig. 3H) (182). These pUL13 activities are important for viral replication, as deletion of pUL13 reduces viral titers. Interestingly, overexpression of pUL13 alone is sufficient to increase the compactness of cristae structure. Mass spectrometry analysis further demonstrates that pUL-13-YFP-HCMV interacts with components of CII, CIII, and CIV. In HCMVΔUL13 infected cells, protein levels for CI, CIV, and CV subunits are decreased (182). Consistently, previous observations showed HCMV increases OXPHOS (183).

Although it is established that alterations in ETC function occur during viral infection, details of structural changes at the individual complex level that result in functional alterations are only starting to emerge (166, 184). Historically, ETC complexes were viewed as static entities that operated as individual complexes. Data have emerged that the individual ETC complexes, from yeast to man, form higher-order quaternary structures with defined stoichiometries termed supercomplexes (60, 185–187). For example, CIV may form dimers, and CI, CIII (2 complexes), and CIV may assemble to form a structure termed the respirasome. SCs are thought to increase OXPHOS efficiency and decrease ROS production (60, 186); however, their physiological relevance is incompletely understood. Uniquely, SCs have been proposed to play structural roles. For example, CV homodimers (CV_2_) appear important for IMM bending and cristae formation (188).

A link between SC dynamics and immune defenses is suggested by newer work. For example, methylation-controlled J protein (MCJ) is a host negative regulator of SC formation (189). Deletion of MCJ in CD8-positive cytotoxic T-cells enhances mitochondrial respiration, which is associated with increased clearance of influenza infection. These findings support a protective role for SCs during viral infection (189). Interestingly, MISTR factors, which are described above, are implicated in regulating SC structure. MISTR1/NDUFA4 interfaces with CIV on the known surface that interacts with the other complexes (55). Proteomics analysis indicates that MISTRAV/C15orf48, the MISTR1/NDUFA4 paralog, interacts with CI, CIII, and CIV (190). Both MISTR1/NDUFA4 and MISTRAV/C15orf48 are differentially regulated by immune signals with MISTRAV/C15orf48 being an interferon-stimulated gene (52, 77). Notably, both are also encoded by viruses and both display signatures of positive selection in primate genomes characteristic of repeated targeting by pathogen-encoded inhibitors (52). MISTR1/NDUFA4 and MISTRAV/C15orf48 can replace each other, in terms of presence/absence, in specific ETC complexes during inflammatory conditions as demonstrated by Blue-Native PAGE analysis (59). This swap in IL-1β-treated cells and during RNA virus infection likely contributes to the observed subsequent changes in mitochondrial membrane potential and mtROS production (59). The mechanism by which replacement of cellular MISTR factors in the ETC modulates host defenses remains an open question. Equally important is how MISTR virologs counteract their functions, especially given three independent transfers of host MISTR factors to viruses (52) that infect hosts separated by more than a billion years of evolution. We hypothesize that cellular MISTR factors like MISTRAV alter ETC composition, especially SCs, early in infection to license immune defenses executed by mitochondria (Fig. 3I).

Although reports describing roles for SCs in regulating host defenses are scant, at least one study highlights bacteria-triggered changes in SCs (191). In this study, mouse macrophages—BMDMs and peritoneal macrophages—were infected with Gram-negative bacteria (E. coli and S. enterica). Recognition of the bacteria through TLR- and NLRP3-dependent pathways decreased levels of CI and CI-containing SCs. Functionally, reduced activity of CI was accompanied by increased activity of CII (191). Future research will work out if ETC composition has key defense functions in immune and nonimmune cells during viral infection.

The cross talk between immunometabolism and antiviral responses is now an appreciated paradigm. Metabolism is also accepted to impact host tolerance to pathogens. Indeed, metabolic diseases like diabetes are often considered major comorbidities for many types of viral infection (192), including SARS CoV-2 (193). Accordingly, understanding metabolic regulation in antiviral defense may lead to innovative therapies for infectious disease. Likewise, virologs of cellular metabolic effectors may provide novel insights into vulnerabilities in circuitry that shape metabolic output.

## CONCLUSIONS AND PERSPECTIVES

As discussed here, mitochondrial activities continue to be associated with the biology of virus infection. Key roles for certain mitochondrial functions are further accentuated by documented viral-encoded antagonists such as virologs. While the mitochondrial genome displays a limited coding capacity (13 protein-coding ORFs), more than 1,000 nuclear-encoded proteins have evidence for mitochondrial localization under homeostatic conditions (66, 67). We expect that moving forward, additional roles for host mitochondrial factors during infection will emerge. Reasonably, the functions of poorly characterized host-encoded factors may become more evident when the biology is considered using a framework based on signatures associated with pivotal host–virus interactions (46, 52). Given insights from host defense and the regulation of mitochondrial functions by cytoplasmic factors, it is fair to assume that the mitochondrial proteome is larger and more dynamic than appreciated. A contributing facet may be the relocalization of other cellular factors to mitochondria in response to infection as a means to establish and regulate immune defenses. An interesting dimension to consider as a mechanism to accomplish localization to and from mitochondria is isoform switching. Namely, mitochondrial localization signals may be gained or lost in isoforms by alternative mRNA splicing (194) as an adaptive move (195–198) or usage of downstream start codons. Similar principles may also be applicable to viral effectors. The characterization of changes in the mitochondrial proteome during infection will be driven, in large part, by proteomics-driven approaches (199, 200). Likewise, novel virologs for host mitochondrial factors will complement future studies and guide the identification of new battlegrounds at this organelle (52, 53). Importantly, evolutionary innovations in virologs, relative to their host counterpart, will be extremely useful in dissecting cellular functions.

Cell culture studies undoubtedly have been powerful in defining many aspects of known biology, yet the impact of different nutrients in cell culture media on mitochondrial functions during virus infection in cultured cells is largely unexplored. However, it has been established that replacing glucose with galactose in media serves to reprogram uninfected cultured cells from glycolysis to oxidative phosphorylation (201, 202). The functional significance of reprogramming to OXPHOS in culture is demonstrated by the increased susceptibility of galactose-grown cells to mitochondrial toxins (201, 203). In addition, the galactose “trick” is commonly used as a means to identify novel regulators of mitochondrial functions, particularly in genetic screens (204). Notably, cells with increased respiration have higher levels of ROS, which as discussed above, influences host defenses in a variety of ways. Likewise, the levels and production of specific metabolites, which serve as donors for enzymes that mediate posttranslational modifications, can have a profound effect on diverse cellular activities and physiology (205). Recent advances in the development of more physiologically relevant medias (206–209), which are now commercially available, will assist in clarifying mechanisms and the functional significance of mitochondrial interfaces during infection. Relatedly, advances in genome engineering, particularly CRISPR (210), will allow the generation of new *in vivo* models in mice and novel exotic animals (211).

Over the coming years, we suspect that many textbook activities associated with mitochondria will be revisited and perhaps even reimagined through studies of host–virus interfaces. The fact that mitochondria are such a hot spot during pathogen infection is not altogether surprising when considering that this entire organelle is a shell of a former organism. Although now extinct, the ongoing battle between extant pathogens and hosts at this site may shed light on pivotal adaptations that occurred for modern complex cells to co-opt an energy-rich prokaryote.
